# Peer Review Week 2020: *Eurosurveillance* thanks all COVID-19 reviewers for their amazing work

**DOI:** 10.2807/1560-7917.ES.2020.25.38.2009242

**Published:** 2020-09-24

**Authors:** Eurosurveillance editorial team

**Affiliations:** 1 European Centre for Disease Prevention and Control (ECDC), Stockholm, Sweden

**Keywords:** peer-review

In this issue coinciding with the 2020 Peer Review Week, we publish the 105th scientific article on coronavirus disease (COVID-19). 

We take this opportunity to express our sincere gratitude to all the fantastic peers who have helped us review these manuscripts, despite your own high workloads and often on short notice. The theme of this years’ peer review week is trust in peer review and particularly in the context of the COVID-19 pandemic we put our trust in scientists and public health experts who support us with their expertise and time. 

Your input is essential in safeguarding the quality and usefulness of published science on this fast-moving topic.

We cannot do this without you.

Thank you!

From the *Eurosurveillance* editorial team

to all who have reviewed COVID-19 articles for us thus far:

Muna Abu Sin; Mary Adams; Cornelia Adlhoch; Jan Albert; Sheikh Taslim Ali; Franz Allerberger; Erik Alm; Christian Althaus; Maria an der Heiden; Matthias an der Heiden; Nick Andrews; Alberto Antonelli; Tommi Asikainen; Barry Atkinson; Erik Aurell; Sabrina Bacci; Agoritsa Baka; Sooria Balasegaram; Cyril Barbezange; Ian Barr; Denis Bauer; Gabrielle Beaudry; Justus Benzler; Isha Berry; Marianne BESNARD; Philippe Beutels; Hubert Blain; Nicki Boddington; Pierre-Yves Boelle; Magnus  Boman; David Boulware; Lisa Brouwers; Mia Brytting; Udo Buchholz; Nicholas Bundle; Thierry Burnouf; Karen Burns; Valeria Cagno; Worth Calfee; Gail Carson; Geraldine Casey; André Charlett; Remi Charrel; Nikoloz Chkhartishvili; Eun Hwa Choi; Gerardo Chowell; Daniel Chu; Bruno Ciancio; Hannah Clapham; Vittoria Colizza; Alex Cook; Samuel Cordey; Victor Corman; Laura Cornelissen; Benjamin Cowling; Natasha Crowcroft; Gavin Dabrera; Niklas Danielsson; Helena de Carvalho Gomes; Sara Dequeker; Jean-Claude Desenclos; Simona Di Giambenedetto; Sabine Diedrich; Michaela Diercke; Joakim Dillner; Jennifer Dowd; Timothee Dub; Dominic Dwyer; Isabella Eckerle; Michael Edelstein; Martin Eichner; David Elliman; Theresa Enkirch; Daniel Faensen; Darryl Falzarano; Bastian Fischer; David Fisman; Per Follin; Anders Fomsgaard; Ivo Foppa; Frode Forland; Anne Fouillet; Gary Garber; Raphael Gaudin; Marius Gilbert; Rosina Girones; John Glasser; Céline Gossner; Magnus  Gottfredsson; Margrethe Greve-Isdahl; Jan Gunst; Sonja Hansen; Heli Harvala; Hans Hirsch; Boris Hogema; Susan Hopkins; Rachel Hoyles; Anette Hulth; Michael Höhle; Giuseppe Ippolito; Esma Islamaj; Angela Iuliano; Eric A Jensen; Kari Johansen; Helen Johnson; Pernille Jorgensen; Charlotte S. Jørgensen; Sarah Kada; Kamran Kadkhoda; Günter Kampf; Anu Kantele; Tommi Karki; Alyson Kelvin; Marie E. Killerby; Anke Kohlenberg; Rolf Kramer; Florian Krammer; Gerard Krause; Tyra Krause; Annelies Kroneman; Adam Kucharski; Tanja Kuchenmuller; Kin On Kwok; Filippo Lagi; Amparo Larrauri; Eric Lau; Quentin Le Hingrat; Jason J Leblanc; Jonggul Lee; Clara Lehmann; Tinne Lernout; Kathy Leung; Marc Lipsitch; Yang Liu; Nicola Low; Xufei Luo; Theodore Lytras; Emma Löf; Pablo M De Salazar; Kristine Macartney; Ian Mackay; Gkikas Magiorkinis; Cecile Magis-Escurra; Benjamin Maier; Michal Mandelboim; Jean-Michel Mansuy; Gerald McInerney; Lucy McNamara; Adam Meijer; Stefano Merler; Benjamin Meyer; Jean Millet; Kenji Mizumoto; Chris Ka Pun Mok; Yamir Moreno; Shila Mortensen; Joël Mossong; Vincent Munster; Michelle Murti; Mette Myrmel; Nathalie Nicolay; Jens Nielsen; Jessica Nihlén Fahlquist; Paulo Nogueira; Hanna Nohynek; Norbert Nowotny; Dominik Sebastian Nörz; Lulla Opatowski; W Paget; Takis Panagiotopoulos; Marcus Panning; Suzan Pas; Lara Payne; Richard Pebody; Pasi Penttinen; Ranawaka A P M Perera; Martin Petric; Anastasia Pharris; Gorben Pijlman; Mateusz Plucinski; Chiara Poletto; Mario Poljak; Leo Poon; Andrea Pugliese; Joan Puig-Barbera; Filip Raciborski; Barbara Rath; Raffaella Ravinetto; Chantal Reusken; Ute Rexroth; Giovanni Rezza; Emmanuel Robesyn; Joacim Rocklöv; Angela Rose; Maiken Rosenstierne; Marc Saez; Linda Saif; Cristiano Salata; Gloria Sanchez; Gianpaolo Scalia-Tomba; Samuel Scarpino; Jonas Schmidt-Chanasit; Ilan Schwartz; Ettore Severi; Anita Shah; Yehuda Shoenfeld; Carlo Signorelli; Andreas Sing; Anika Singanayagam; Mary Anissa Sinnathamby; Kristian Soltesz; Gianfranco Spiteri; Heribert Stoiber; Sunita Sturup-Toft; Kanta Subbarao; Carl Suetens; Makoto Takeda; Karin Tegmark-Wisell; Anders Tegnell; Peter Teunis; Craig Thompson; Robin Thompson; Stacy Todd; Alma Tostmann; Ramona Trebbien; Tim Tsang; Gro Tunheim; Helmut Uphoff; Palle Valentiner-Branth; Birgit van Benthem; Kees. C. van den Wijngaard; Wim van der Hoek; Edward van Straten; Guido Vanham; Philippe Vanhems; Olli Vapalahti; Lasse Vestergaard; Cécile Viboud; Russell M Viner; Leo Visser; Silja Vöneky; Jacco Wallinga; Tobias Welte; Tobias Welte; Annelies Wilder-Smith; Emma Wiltshire; Christian Winter; Katja Wolthers; Joseph Wu; Roman Wölfel; Andrea Würz; Takuya Yamagishi; Dong Keon Yon; Barnaby Edward Young; Ioannis Zabetakis; Maria Zambon; Lorenzo Zammarchi; Philipp Zanger; Robert Zangerle; Xu-Sheng Zhang.

We apologise to any reviewers whose names we may have missed.

